# Breast Cancer—How Can Imaging Help?

**DOI:** 10.3390/healthcare10071159

**Published:** 2022-06-22

**Authors:** Roxana Iacob, Diana Luminita Manolescu, Emil Robert Stoicescu, Antonio Fabian, Daniel Malita, Cristian Oancea

**Affiliations:** 1 Department of Radiology and Medical Imaging, ‘Victor Babes’ University of Medicine and Pharmacy Timisoara, 300041 Timișoara, Romania; roxana.iacob@umft.ro (R.I.); stoicescu.emil@umft.ro (E.R.S.); antoniofabian9319@yahoo.com (A.F.); malita.daniel@umft.ro (D.M.); 2 Center for Research and Innovation in Precision Medicine of Respiratory Diseases (CRIPMRD), ‘Victor Babeș’ University of Medicine and Pharmacy, 300041 Timișoara, Romania; oancea@umft.ro; 3 Research Center for Pharmaco-Toxicological Evaluations, ‘Victor Babes’ University of Medicine and Pharmacy Timisoara, 300041 Timișoara, Romania; 4 Department of Pulmonology, ‘Victor Babes’ University of Medicine and Pharmacy, 300041 Timișoara, Romania

**Keywords:** breast cancer, imaging, imaging techniques, mammography, breast ultrasound, breast MRI

## Abstract

Breast cancer is the most common malignant disease among women, causing death and suffering worldwide. It is known that, for the improvement of the survival rate and the psychological impact it has on patients, early detection is crucial. For this to happen, the imaging techniques should be used at their full potential. We selected and examined 44 articles that had as subject the use of a specific imaging method in breast cancer management (mammography, ultrasound, MRI, ultrasound-guided biopsy, PET-CT). After analyzing their data, we summarized and concluded which are the best ways to use each one of the mentioned techniques for a good outcome. We created a simplified algorithm with easy steps that can be followed by radiologists when facing this type of neoplasia.

## 1. Introduction

Breast cancer (BC) represents one of the most common forms of malignancy, along with colon and lung cancer, being a leading malignant tumor and the primary cause of death and suffering among women worldwide [1,2,3,4].

Studies show that it can be determined by both genetic and non-genetic factors, such as environment, age, and reproductive history, and can have various types of manifestation [5,6,7]. Based on the histological and molecular findings, breast cancer has been classified into three categories:

1. BC expressing hormone receptor (estrogen or progesterone);
2. BC expressing human epidermal receptor 2;
3. Triple-negative breast cancer, not expressing any of the above-mentioned receptors [7,8].

To improve the survival rates, early detection of this neoplasm is essential, since breast cancer with no metastases is likely curable [1,9]. Unfortunately, breast cancer with distant metastases is currently considered incurable, even with the new advances in current therapies [10].

For a better outcome regarding the patients’ health, a multidisciplinary team, including radiologists, should be implemented to participate in all steps between breast cancer suspicion and posttherapy surveillance in confirmed patients [1]. Advances in breast imaging are known to influence the prognosis and treatment of breast cancer patients in a beneficial way [11]. Imaging techniques are used for diagnostic approaches, monitoring disease progression, and treatment response [3]. The most used imaging method in detecting breast cancer is mammography, being the method of choice for most radiologists [12]. Even though it has high sensitivity and specificity, its advantages are attenuated in women with dense breasts. Breast density is subjectively measured by the radiologist, comparing the radio-opaque parenchyma to the radiolucent fatty tissue [5,12]. In these cases, breast ultrasound (US) is an adjuvant technique to mammography, one of the most common imaging methods for diagnosis and monitoring [5,12]. With these techniques, a BI-RADS (Breast Imaging Reporting and Data System) score from 1 to 6 is given to suspicious lesions on each breast, which dictates the following steps [13,14]. This score was implemented to reduce the variability among radiologists when creating reports, regarding not only mammography but also ultrasound and MRI [15,16].

Another important tool for monitoring is the MRI and additionally the use of contrast, which improves the method’s sensibility [12]. US-guided biopsy has a lower risk of complications compared to surgery and can evaluate non-palpable breast lesions [17]. Furthermore, it can improve the quality of life, being a less invasive procedure than surgery [3].

This literature review presents the algorithm and intends to show the most advantageous ways to use and link the existing imaging methods to improve both the management and outcome of patients with breast cancer, from suspicion and diagnosis to posttherapy surveillance and follow ups. The main imaging techniques and their clinical applications were summarized to find the best way to manage breast cancer patients.

## 2. Materials and Methods

The studies included in this review were chosen according to the PRISMA (Preferred Reporting Items for Systematic Reviews and Meta-Analyses) guidelines. The present review is based on bibliographic searches in PubMed database, Google Scholar, and Scopus, using both manual search and MeSH terms (on PubMed). Articles related to breast cancer imaging, published in the last 5 years were selected. The most relevant articles were chosen based on their title, the information given in their abstract, and a quick view of the complete manuscript. We excluded articles written in a language other than English, publications that had only the abstract available, and duplicates.

The search and selection were conducted in January 2022, by two radiologists with experience and knowledge in breast imaging. As a first step, we manually searched articles based on the keyword “breast cancer” and each of the imaging methods alone: “mammography”, “tomosynthesis”, “ultrasound”, “elastography”, “MRI”, “ultrasound-guided biopsy”, and “PET-CT”. Additionally, using the MeSH term option available in PubMed, we conducted another search with the following terms: ((“Breast Neoplasms” [Mesh]) AND “Diagnosis” [Mesh]) AND “Disease Management” [Mesh].

For better management and planning of the review, all the selected articles were incorporated in a Microsoft Excel table, with the following columns: title, authors, used imaging method, year of publication and journal, type of publication, and keywords.

We selected the most relevant articles for each imaging method mentioned above (between 6 and 14 articles per imaging tool), focusing on the best use of each imaging technique in all the steps a breast cancer patient undergoes—suspicion, diagnosis, treatment, and surveillance. In total, 44 articles met all the criteria and were selected for the literature review. We discussed their most important data and grouped the results as follows:

1. Mammography and tomosynthesis in BC;
2. Ultrasound and elastography in BC;
3. MRI in BC;
4. Ultrasound-guided biopsy in BC;
5. PET-CT and PET-MRI in BC.

**Figure 1:**
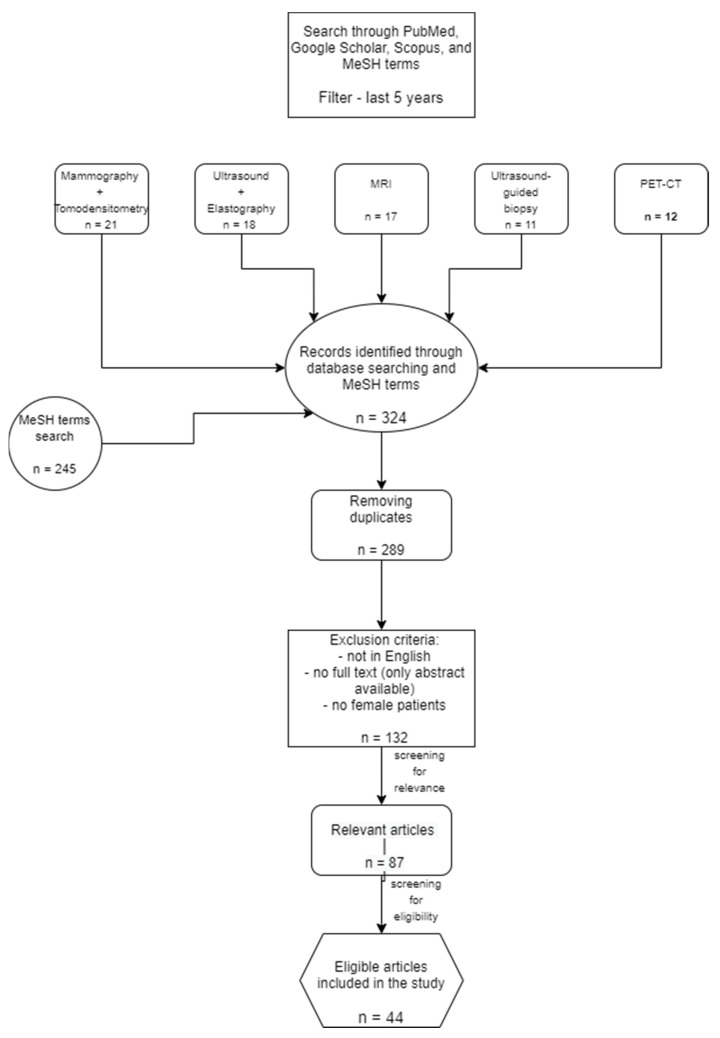
PRISMA diagram with the selected articles.

The diagram below summarizes the steps followed for selecting the articles included in the review (Figure 1).

## 3. Results

### 3.1. Mammography and Digital Tomosynthesis in Breast Cancer

Most experts suggest that mammography screening decreases the mortality rate of breast cancer by up to 33%, but this also leads to overdiagnosis of neoplasia that would not have caused morbidity or death [18]. Some lesions may look suspicious on mammography but are proven to be benign [19]. Lately, mammography has substantially improved, raising its sensitivity and specificity, through the additional use of digital breast tomosynthesis—3D mammography—images obtained at the same time as 2D digital mammography [18,20]. The addition of tomosynthesis increases the detection of early invasive breast cancer by detecting small lesions and distortion [21,22]. Given the fact that tomosynthesis used in addition to mammography doubles the radiation exposure, a cautious indication must be taken into consideration [18,21].

Even though diagnostic mammography has a high sensitivity, its specificity is relatively low, especially in women with dense breasts, and therefore, ultrasound is usually additionally performed [17,23].

Another imaging method derived from mammography is contrast-enhanced mammography (CEM), which uses iodinated contrast agents for the easier detection of tumor growth due to angiogenesis [24]. This imaging tool gives not only morphologic information but also data about the tumor’s physiology [23]. Compared to standard 2D mammography, contrast-enhanced spectral mammography has a higher sensitivity and diagnosis performance, being able to identify neoplasia even in dense breast tissue [24,25]. Even if MRI is the method of choice for breast tumor staging, CEM has a higher specificity and nearly the same sensitivity [24]. For patients with claustrophobia for example or MRI-incompatible implants, CEM is a good alternative [25]. As indications for CEM, the authors note preoperative estimation of disease extent, evaluation of symptomatic patients, or posttreatment [23]. CEM’s disadvantages include allergy to contrast agents and higher radiation dose than mammography [23].

Regarding pregnancy-associated breast cancer, which includes tumors diagnosed during or 12 months after pregnancy, US is the most chosen imaging tool, but when malignancy is suspected, authors note that mammography should exclude multicentric or bilateral cancer, so that the radiation dose for the fetus remains under the critical threshold [26].

### 3.2. Ultrasound and Elastography in Breast Cancer

Ultrasound can be used both in the screening and diagnosis of breast cancer, having superior performance in young women, due to the fact that they have more glandular tissue (dense breasts) than older women [27]. It can assess the orientation, structure, margins, and morphology of the lesions [15]. Compared to mammography, a metanalysis showed that US has a higher sensitivity (81% vs. 65%), but no significant differences in specificity [27]. Unfortunately, some of the studies suggest that the shape of the lesions seen on ultrasound does not correlate with the molecular markers, but hyperechoic and mixed echogenicity has a strong correlation with ER- and PR-tumors [28]. Being fast, repetitive, and non-irradiating, US is becoming the most popular imaging method used for breast cancer diagnosis, studies show [28]. Besides the fact that compared to mammography, US is radiation free, it has higher accuracy in detecting lesions in women with dense breasts, also detecting more invasive cancers than mammography [29]. Some of the authors concluded that the average lesion size detected by mammography was 14.7 mm, while ultrasound could detect cancers with a mean size of 13.5 mm [29]. As a limitation mentioned in most of the studies, US is not able to detect small breast calcifications as mammography does, having no indication for replacing mammography in BC screening [27,29].

Another utility for US is a preoperative examination of the axilla, combined with ultrasound-guided biopsy of suspicious lymph nodes, which resulted in a sensitivity of almost 80% and a specificity of 100% [15,30]. The signs highly associated with lymph node metastases are irregular shape and high color Doppler flow imaging grades [15]. Nevertheless, preoperative US did not change the surgical approach in most of the cases [30].

Additionally, US can be used intraoperatively, mainly for identifying non-palpable lesions and for helping the surgeon obtain satisfactory margins [31]. The authors also concluded that this technique has no risk of complications and gives the surgeon greater precision [31].

In a study published in 2018, Leblond et al. raised the problem of contralateral breast US, resulting in more than 16% of the patients having abnormalities and also suspicious contralateral lymph nodes [32]. This finding changed the therapy for most of the patients [32].

Derived from ultrasonography, elastography has gained popularity in the last few years for breast cancer detection and classification; training is the most important factor regarding this technique [13,33,34]. This imaging technique can measure the stiffness of lesions by measuring the consistency and hardness of a structure, a fact that can differentiate a malign mass (greater stiffness) from a benign one [15,35]. On elastography, malignant lesions appear larger than in US, while benign lesions look smaller [36]. Carlsen et al. conclude that even if elastography has high accuracy in detecting malign breast lesions, it cannot replace US and is not feasible to be used alone, but the combination of these two has really good results [37]. Moreover, if the technique is qualitative, the number of biopsies with negative results can be avoided [36]. For examining axillary lymph nodes, elastography measures the proportion of the hard area, being positive if it is greater than 50%, unlike US, which assesses lymph nodes by diameter, cortical thickness, the status of the hilum, and vascular pattern [38].

There are a few different types of elastography, for example, strain elastography, acoustic radiation force impulse imaging, transient elastography, point shear wave elastography, and shear wave elastography [15]. The most used ones are strain elastography and shear wave elastography. Strain elastography is based on manual repetitive compression with the transducer, and the amount of deformation in the tissue is measured, while shear wave elastography uses acoustic radiation force impulse, measuring the propagation speed of shear waves in the lesion, measuring its stiffness [39]. Elastography improves ultrasound specificity, reducing the need for benign biopsies when used complementary to the conventional US, being a supplementary tool in diagnosing breast cancer, especially in women with dense breast tissue [15,35]. Most of the studies conclude that using multimodal imaging raises the accuracy of breast cancer diagnosis [13].

### 3.3. MRI in Breast Cancer

Studies show that breast MRI has a promising role in breast cancer management and can be used in addition to mammography in diagnosing this type of neoplasia, especially in patients with dense breasts [40,41]. It is demonstrated that MRI has a higher sensitivity in detecting breast neoplasia than mammography and ultrasound (also in women with prostheses), which makes it an effective technique in investigating cancer recurrence [42,43,44,45,46]. Furthermore, it can detect neoplasia at an early stage, compared to mammography or US, being able to differentiate benign lesions from the benign-appearing ones [45,46].

However, most of the studies do not recommend using MRI in breast cancer follow up, but some of them suggest that MRI, US, and mammography are often used complementarily, for a better outcome [42,45]. As risk factors that indicate MRI screening, we mention BRCA mutations or a first-degree relative of a person with this kind of mutation, occult breast cancer—not identified on US or mammography but presenting metastases or bloody nipple discharge [46]. Other authors suggest that MRI should be used for BC screening and also for women with a history of chest radiation at a young age [45].

Schoub et al. conclude that breast MRI’s sensitivity is between 98% and 100%, its specificity is 88%, while the negative predictive value (NPV) is around 100%, being useful in confirming the absence of neoplasia [46]. Other authors note that this imaging tool has a high sensitivity but low specificity for both benign and malign lesions [47]. In addition, the authors note that MRI can identify cancer in the contralateral breast in 3–6% of the cases and in up to 27% of the cases, in the same breast [46]. For evaluating the residual disease after neoadjuvant chemotherapy, MRI is thought to be more accurate than US, mammography, or clinical evaluation, with a sensitivity of 83% [41].

Furthermore, using gadolinium-based contrast agents can improve the detection of angiogenesis, this type of investigation is the base of dynamic-contrast enhanced MRI (DCE-MRI) [40,48]. With the help of this technique, cancer not detected on mammography can be seen, and it is also able to differentiate benign from malign lesions [40,48]. Additionally, MRI can be used for local staging and to diagnose muscular invasion [40]. Studies show that breast MRI is better than the other imaging methods in identifying residual tumors [40]. Unfortunately, some studies conclude that MRI may overestimate or underestimate residual disease, depending on the tumor type [49]. DCE MRI has been also used for the detection of breast cancer, based on the theory that every lesion larger than 2 mm cannot grow without creating new blood vessels [45].

Unfortunately, at present, this imaging technique is not used at its maximum potential because clinicians are not aware of all its benefits [46].

### 3.4. Ultrasound-Guided Biopsy in Breast Cancer

An ultrasound-guided breast biopsy is preferred compared to surgical biopsies, has lower risks and side effects, and is faster than the last-mentioned method [50,51,52]. Additionally, it has high accuracy in detecting metastatic lymph nodes [51]. Even though they are rare, post-biopsy complications have to be mentioned as they include hematoma, infection, allergic or vagal reaction, and even pneumothorax [53]. Most breast ultrasound-guided biopsies of suspicious lesions are benign, which reduces unnecessary surgery [53]. Even though some of the lesions were found by another imaging tool (mammography, MRI), ultrasound is the preferred method when doing the biopsy. For it to be effective, the radiologist should pay attention to punction the same lesion seen on the other imaging modality, as the aspect may vary [53].

Another way to use the US is for guiding breast cryoablation, a minimally invasive technique that can “kill” malignant tissue with cold. It is successful in 85–100% of cases for invasive ductal carcinoma larger than 2 cm and in 100% of tumors under 1 cm and can also be used for fibroadenomas [54].

Regarding biopsies, digital breast tomosynthesis (DBT) vacuum-assisted biopsy is becoming increasingly popular, being able to detect lesions not seen on ultrasound or categorized as occult in US. It is especially good at detecting distortions; it is easy and quick to perform and has a low risk of complications. Authors suggest that it is a good way to establish the diagnosis of suspicious lesions [55].

### 3.5. PET-CT and PET-MRI in Breast Cancer

Unlike mammography, breast ultrasound, and breast MRI, FDG PET-CT has a low sensitivity for primary breast tumors—this method is unable to detect cancers under 1 cm in dimension [56,57]. Even though it is not a feasible method for detecting the local extent of neoplasia, it is a very helpful tool in the systemic staging of the disease [56,57]. Additionally, it can give important information about the primary tumor, with studies showing that the extent of FDG uptake by the tumor cells can differentiate between the cancer grade and subtype [57]. It is also a good alternative for detecting distant metastases [57]. Apart from malignant breast cancer, FDG can be captured also by inflammation, infections, fibroadenomas, and even physiologic tissue during lactation [56].

Although FDG PET-CT is recommended to be used for staging in advanced breast cancer and for the inflammatory type of neoplasia, studies show that it is not recommended for early-stage neoplasia [58,59]. The same review states that, for triple-negative breast cancer, this type of imaging technique has not been sufficiently studied to be recommended [58]. Regarding regional lymph node staging, FDG PET-CT has higher accuracy than other imaging techniques [58]. It was also demonstrated that the use of FDG PET-CT changed the initial staging and therapy and is helpful in the surveillance of neoadjuvant chemotherapy [58,60]. Using PET-CT after neoadjuvant chemotherapy can avoid radical mastectomy, detecting the presence of residual neoplasia better than MRI [61]. For investigating regional nodal recurrences, which is a sensitive indicator of breast cancer survivors, PET-CT has been proven to be an efficient and accurate method [62].

In the past few years, the use of PET-MRI is escalating, being used in the diagnosis, staging, and treatment assessment of BC. It is a quantitative hybrid imaging method that combines metabolic and functional details (PET) with information regarding anatomy and perfusion (MRI) [63].

Both PET-CT and PET-MRI have a high sensitivity in detecting axillary and extra-axillary lymph node metastases, but PET-CT is better for lung metastases, while PET-MRI detects bone and liver secondary determinations better [64].

The advantages and disadvantages of the above discussed imaging methods used for dealing with breast cancer, can be found in the table below (Table 1).

## 4. Discussion

As breast cancer is one of the most common malignancies among women and an important cause of death, this disease is considered to be a public health problem [2]. For a better outcome for the patients with suspicion and diagnosis of breast cancer, very accurate management is necessary. It was demonstrated that early and correct handling of the disease decreases mortality and also makes the journey of a breast cancer patient less painful and harmful. For this to happen, all the disponible imaging techniques should be integrated and used together. As each imaging method has its advantages and disadvantages, it is important to know what the best use is for each of them and to have a kind of “imaging algorithm” for BC, from screening the high-risk patients to posttreatment surveillance of a confirmed BC.

Digital mammography and, recently, 3D tomosynthesis are the main tools used for screening (in women with risk factors) and diagnosis (women with suspicion of breast cancer, for example—those with a palpable mass) [18,21]. Tomosynthesis can also be used for the biopsy of lesions that seem occult on ultrasound [55]. As an obstacle to these imaging techniques, dense breast tissue is mentioned, which can be examined by ultrasound and elastography, which have a better accuracy in this case. Ultrasound can be also used for metastatic lymph node diagnosis and as a part of the operative act—helping surgeons detect non-palpable masses [32]. Breast biopsy, whether guided by US, mammography, or MRI, is the best way to get a certain diagnosis of the tumor type, in order to start the specific treatment [50,51,52].

Breast MRI is the most effective in diagnosing cancer recurrence, and, when contrast agents are added to this technique, it can detect angiogenesis. It can also detect lymph node metastases and helps with staging advanced breast cancer, as well as FDG PET-CT, which has high accuracy and can also be used for surveillance of the neoadjuvant chemotherapy [42,57].

All these imaging techniques have their advantages but also some inherent disadvantages, which is why their features and particularities need to be very well known by radiologists. For the best outcome for a patient diagnosed with breast cancer, at each stage (from suspicion and diagnosis to posttreatment surveillance and disease recurrence), the optimal imaging tool should be chosen. Nevertheless, there is no correct method or steps to follow when dealing with this disease, because each patient has their own particularities and development of neoplasia. Thus, the radiologists have to decide on their own how to juggle these techniques and determine the best time to use each one of them.

For better management of this disease, and based on the articles chosen in this review, we developed an algorithm using the imaging techniques. First of all, for screening and diagnosis of breast cancer, mammography should be used for patients over 40 years old or even younger, who have a high risk of cancer development. If these criteria are not applicable, ultrasound is a good alternative both in screening and diagnosis. MRI plays an important role in women with dense breasts or implants and has a high sensitivity in detecting neoplasia.

For a diagnosis confirmation, ultrasound-guided breast biopsy and also biopsy using other imaging techniques, such as mammography or MRI are preferred over open surgery. Besides its role in confirming the diagnosis, ultrasound can be used for the cryoablation of small malign masses.

Although PET-CT is not as sensitive as the above-mentioned imaging methods in detecting the primary tumor, it is used with great results in detecting the tumor extension and staging the disease and can also be used for posttreatment surveillance and follow up.

**Figure 2:**
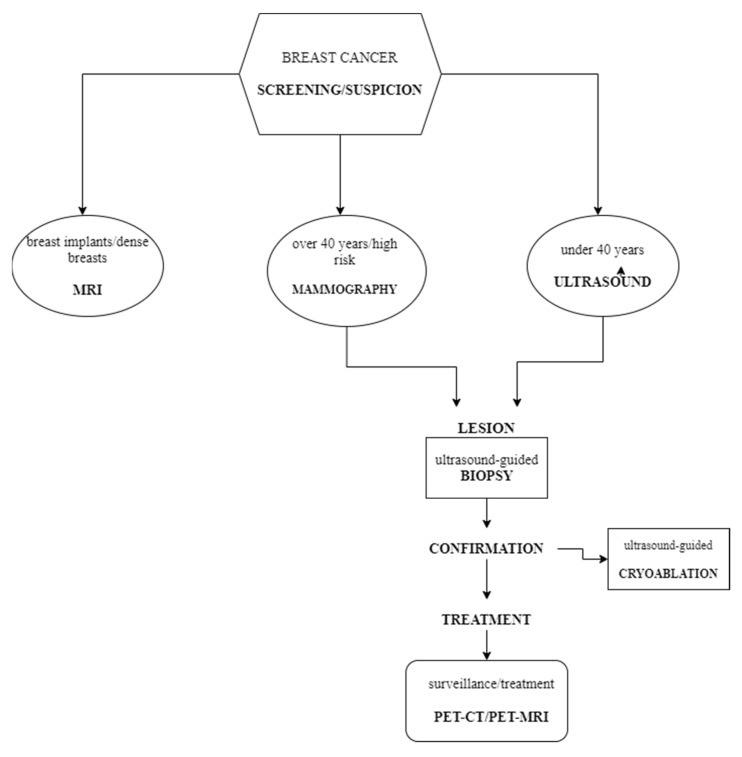
The algorithm for using imaging methods in the breast cancer management.

In the figure below, we summarized the steps to be followed from screening to the surveillance of breast tumors, focused on the best way to use the most popular imaging techniques (Figure 2). Based on the information gathered from all of the studied articles, the following scheme is the most used “imaging algorithm” when dealing with breast cancers.

## 5. Conclusions

Although each of the above-mentioned methods has an important role in the management of breast cancer patients, they should all be integrated. The studied articles show that, for the best outcome for the patient, we have to take advantage of all the imaging tools at their full potential. Mammography and 3D tomosynthesis should be used in screening and primary diagnosis, with the help of ultrasound. Furthermore, ultrasound can be used along with MRI and PET-CT for the staging of the tumor and also intraoperatively. MRI can help with posttherapy surveillance as well as PET-CT. Finally, an ultrasound-guided biopsy can be used for avoiding unnecessary surgery.

All in all, the correct use of the above-mentioned imaging techniques and all their newly derived methods can help with a better outcome for patients diagnosed with breast cancer.

## Figures and Tables

**Table 1:** Advantages and disadvantages of the imaging tools.

Imaging Technique	Advantages	Disadvantages
Mammography	Screening—reduces mortality by up to 33% [18]; high sensitivity [17].	Ionizing radiation [18]; low specificity in dense breasts [23].
Tomosynthesis	Detection of early invasive breast cancer [18,20]; can detect small lesions and distortions [18,20].	Doubles the radiation exposure compared to mammography [18,21].
Contrast-enhanced mammography	Higher sensitivity and diagnosis performance compared to mammography [24,25]; can identify cancer in dense breasts [25]; tumor staging (for patients with claustrophobia or MRI-incompatible implants) [25]; preoperative estimation of disease extent [23].	Allergy to contrast agents [23]; higher radiation dose than mammography [23].
Breast ultrasound	Both in screening and diagnosis [27]; helpful in dense breast tissue; non-irradiating [27]; repetitive [27]; preoperative examination of the axilla [30].	Does not detect small breast calcifications [27,29].
Breast MRI	Higher accuracy in detecting lesions in women with dense breasts [40]; early detection of cancer recurrence [42]; evaluation of residual disease after neoadjuvant chemotherapy [41].	Low specificity for both benign and malign lesions [47]; women with claustrophobia [25]; implants or other materials not compatible with MRI [25].
Ultrasound-guided breast biopsy	Confirmation of neoplasia and its cellularity type [50,51,52]; lower risks and side effects compared to surgical biopsies [51]; high accuracy in detecting metastatic lymph nodes [51]; reduces unnecessary surgery [53].	Associated risks: bruising and swelling, infection, bleeding [51,52].
PET-CT	Systemic staging of the disease [56]; detection of distant metastases [57]; after neoadjuvant chemotherapy, can avoid radical mastectomy by detecting the presence of residual neoplasia better than MRI [61].	Low sensitivity for primary breast tumors [56,57]; not able to detect cancers under 1 cm [57].

## Data Availability

Not applicable.
